# In Vitro Fracture Strength of Primary Canine Teeth Reinforced With Prefabricated and Customized Fiber‐Reinforced Post Systems

**DOI:** 10.1002/cre2.930

**Published:** 2024-09-18

**Authors:** Faeze Behzadpour, Nilgoon Pasdar, Ghazaleh Ahmadizenouz, Ali Bijani

**Affiliations:** ^1^ Student Research Committee Babol University of Medical Sciences Babol Iran; ^2^ Dental Materials Research Center, Health Research Institute Babol University of Medical Sciences Babol Iran; ^3^ Oral Health Research Center, Health Research Institute Babol University of Medical Sciences Babol Iran; ^4^ Social Determinants of Health Research Center, Health Research Institute Babol University of Medical Sciences Babol Iran

**Keywords:** canine, customized, fracture strength, prefabricated

## Abstract

**Objective:**

The demand for esthetics has increased in today's world and most parents prefer to preserve their children's primary anterior teeth until their natural exfoliation. However, an intracanal post is required to provide retention for reconstruction of severely damaged anterior teeth due to caries or trauma. Various materials and methods may be used for the fabrication of intracanal posts. This study assessed the fracture strength and fracture mode of primary canine teeth reconstructed with prefabricated and customized polyethylene and glass fiber posts.

**Materials and Methods:**

This in vitro study evaluated 60 extracted primary canine teeth in four groups (*n* = 15). After pulpectomy and post space preparation with 4 mm depth, composite resin post, prefabricated glass fiber post (Whitepost), customized glass fiber post (Interlig), or customized polyethylene fiber post (Ribbond) were placed in the root canals to provide retention, and the tooth crown was restored with bulk‐fill composite resin. The fracture strength was then measured in a universal testing machine. The fracture mode was also evaluated visually.

**Results:**

The mean fracture strength was 22.45 ± 5.06, 33.10 ± 8.5, 30.20 ± 7.33, and 32.61 ± 5.73 N/mm^2^ in the composite resin post, Whitepost, Interlig, and Ribbond groups, respectively. The fracture strength was significantly lower in the composite group than in the remaining three groups (*p* = 0.000). No other significant differences were found (*p* > 0.05). Also, no significant difference was observed among the study groups in the fracture mode (*p* = 0.241).

**Conclusion:**

The composite resin post yielded a significantly lower fracture strength than the prefabricated and customized glass and polyethylene fiber posts, but the fracture mode was not significantly different among the four groups.

## 1 Introduction

Dental caries is a chronic, irreversible, and multifactorial disease that may develop due to nocturnal feeding, poor oral hygiene habits, and excessive consumption of cariogenic foods in children. Parents often seek dental treatment for their children when the teeth are severely damaged, making it difficult for the dentist to properly restore them (Arumugam and Sundaramurthy 2021). Preservation and restoration of primary teeth are essential for children's well‐being, facial esthetics, speech, and prevention of destructive oral habits (Ghazawy and Badran 2018).

Reconstruction of severely damaged primary anterior teeth is a challenge due to insufficient residual tooth structure for bonding and poor cooperation of most children (Mital et al. 2021). The conventional simple restorative procedures are not often successful in the restoration of severely damaged teeth with insufficient residual tooth structure and often lead to tooth extraction (Arumugam and Sundaramurthy 2021). However, early loss of primary anterior teeth can lead to several problems, including abnormal tongue position and development of abnormal tongue habits (such as tongue thrusting), speech disorders such as difficulty in pronouncing consonants (t, d, s, sh, ch) and labial sounds (f, v), and mouth breathing habit (Thakur and Ramarao 2019; Mehra et al. 2016; Chandra and Idris 2014; Malakar, Tripathi, and Rahat 2019). In severely damaged anterior teeth with pulp involvement, pulpectomy and subsequent placement of post and core restoration can serve as a favorable treatment option (Arumugam and Sundaramurthy 2021).

An intracanal post is indicated for primary teeth that have lost over 50% of their coronal structure and have a minimum of 1 mm of tooth structure remaining above their gingival margin (Arumugam and Sundaramurthy 2021). Placement of an intracanal post has several advantages in primary teeth; it provides retention for the core, supports the final restoration, improves the retention of strip crowns, increases the resistance to mechanical forces, and enhances the function and esthetic appearance of the tooth (Arumugam and Sundaramurthy 2021).

The characteristics of an ideal intracanal post include optimal biocompatibility, good compatibility with the internal dentin structure to provide core support, optimal core resistance without additional stress application to the tooth structure, high resistance, and no interference with the eruption of permanent successors (Arumugam and Sundaramurthy 2021).

Due to the physiological resorption and exfoliation of primary teeth, intracanal posts are only placed in the coronal third (3 mm) of the root canal to provide retention for coronal reconstruction of pulpectomized primary anterior teeth. It should be noted that the coronal third is the strongest part of the root to withstand functional stresses (Malakar, Tripathi, and Rahat 2019; Morawala et al. 2017; Seraj et al. 2015; Oskouei, Jafarabadi, and Vafaei 2016; Sholapurmath and Anand 2010; Ansari et al. 2017).

Intracanal posts are fabricated from different materials such as nickel–chromium alloy, carbon, glass, and polyethylene; among these, glass and polyethylene fiber posts are the most common due to their optimal fracture resistance and durability in endodontically treated and pulpectomized teeth (Mangoush et al. 2017; Eapen et al. 2017; Mittal et al. 2018). In addition, intracanal posts may be divided into two groups, namely, prefabricated and customized posts. Prefabricated posts are available in various sizes and may require preparation of the root canal space. However, customized posts can be adapted to the canal space due to their formability, thus minimizing the need for further removal of tooth structure (Arumugam and Sundaramurthy 2021; Thakur and Ramarao 2019).

Each type of fiber post has its own characteristics and advantages; thus, knowledge about their advantages and limitations can help dental clinicians select the best fiber‐reinforced composite for each clinical scenario (Morawala et al. 2017). A systematic review in 2017 by Mangoush et al. (2017) showed the superiority of fiberglass over polyethylene posts in 79.3% of the studies. However, Shah et al. (2021) in a systematic review on permanent teeth in 2021 reported that polyethylene fiber posts had a higher fracture resistance than glass fiber posts.

Despite numerous case reports available on primary tooth restorations, information is limited about the characteristics of post‐retained restorations, especially on different types of customized posts (Malakar, Tripathi, and Rahat 2019). Additionally, controversy exists in the literature regarding the preferred type of fiber (glass or polyethylene) for post and core restoration of primary anterior teeth. Fracture mode is another important issue in restored teeth since it shows the quality of bonding of the material to the tooth structure and repairability of the tooth in case of fracture from a clinical point of view (Morawala et al. 2017). Thus, this study aimed to compare the fracture strength of customized and prefabricated glass and polyethylene fiber posts in severely damaged primary canine teeth. The fracture mode was also evaluated in the present study.

## 2 Materials and Methods

The study protocol was approved by the Ethics Committee of Babol University of Medical Sciences (code: IR.MUBABOL.HRI.REC.1401.119).

### 2.1 Specimen Preparation

**Figure 1:**
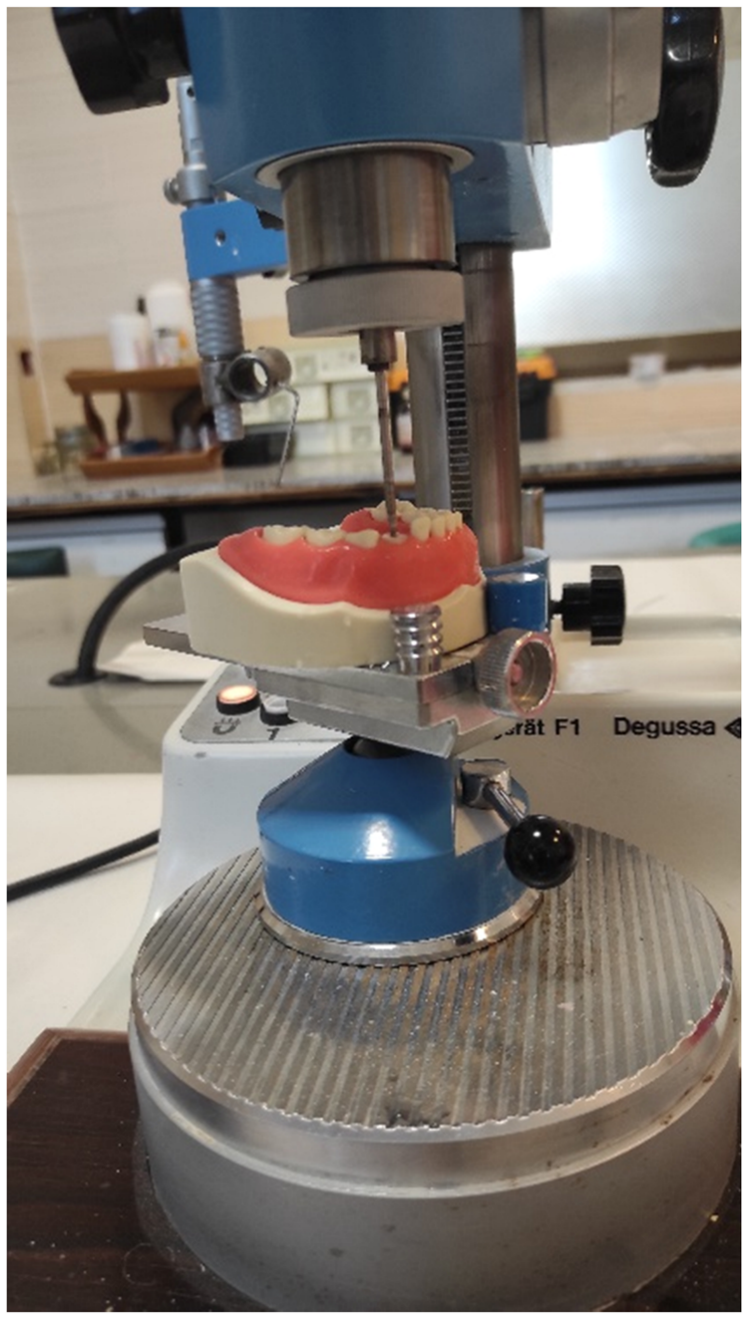
Standardization of the post space using a milling machine.

Sixty maxillary primary canine teeth with intact roots extracted for orthodontic reasons (in cases with severe space shortage requiring serial extraction) were selected for this study. After receiving informed consent from the subjects, the teeth were cleaned and immersed in 0.5% chloramine‐T solution for 1 week and were then stored in distilled water at 4°C until use. The teeth were sectioned horizontally 1 mm above their cementoenamel junction (CEJ) with a diamond bur in a high‐speed handpiece and any remaining pulp chamber roof was removed. Then, the root canals were prepared to #45 K‐file (Mani, Japan) 1 mm short of the apex. Subsequently, the root canals were dried with paper points and filled with calcium hydroxide paste and iodoform (Metapex; META BIOMED Co. Ltd., Korea). After removing 4 mm of Metapex filling material from the coronal part of the canal, the orifice was sealed with 1 mm of polycarboxylate cement (Masterdent Co., USA) to leave a 3‐mm space for the intracanal post. To standardize the post space, all teeth were milled in a milling machine with a #16 carbide round bur with 44.5 mm height (Dia‐Tessin; Switzerland) (Figure 1).

Next, the teeth were randomly divided into four groups (*n* = 15) by block randomization. Afterward, each specimen was coded (including the group and the number of specimens).

### 2.2 Bonded Surface Area Calculation

**Figure 2:**
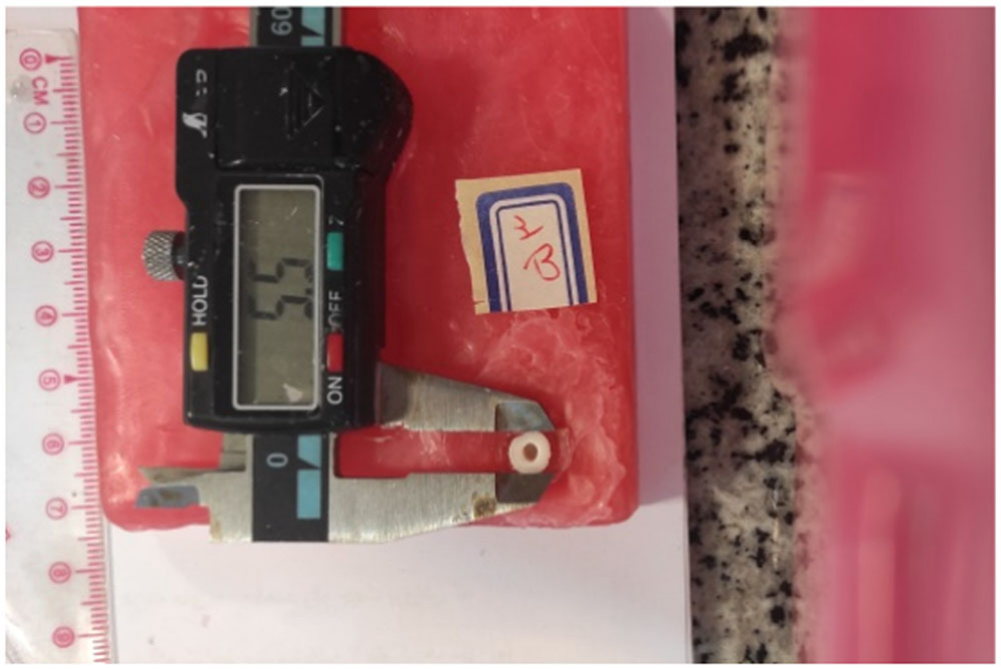
Standard photography of the specimens.

**Figure 3:**
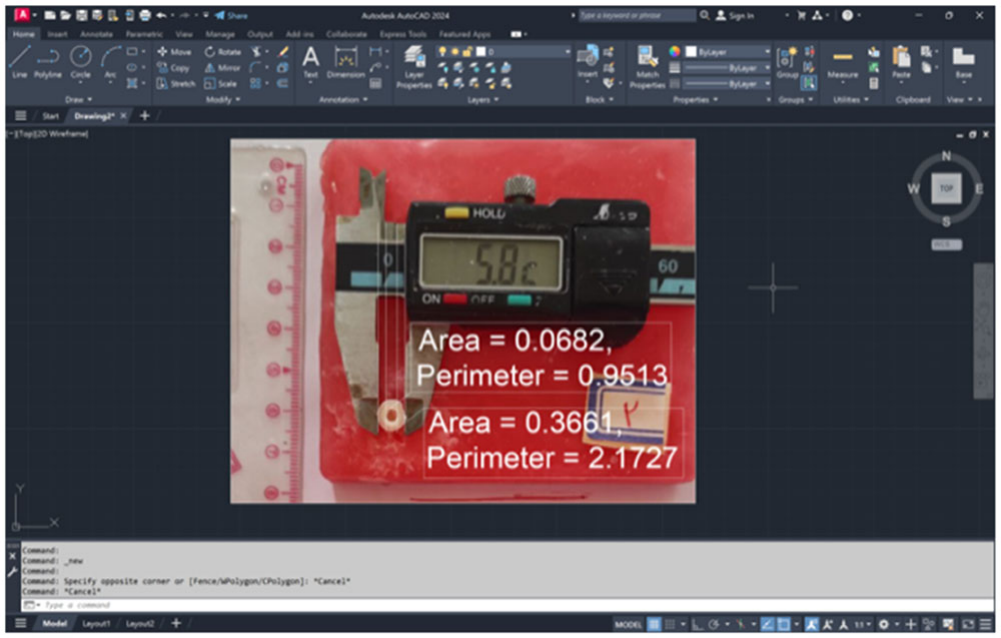
Calculation of the bonded surface area by Autocad software.

Due to the effect of the bonded surface on fracture resistance, standardized photographs were obtained from the specimens at the same distance with a scale on the photographs. Next, the bonded cross‐sectional area of each specimen was calculated at the periphery of the canal and intracanal parts using Autocad 2019 software (Figures 2 and 3).

### 2.3 Post Placement

#### 2.3.1 Group 1: Composite Resin Post

After irrigation, the root canal was dried and 37% phosphoric acid etchant (Condac 37; FGM, Brazil) was applied for 15 s, followed by 10 s of rinsing and then drying with compressed air, allowing the dentin to remain slightly wet. Next, two consecutive coats of a light‐cure bonding agent (Ambar Universal; FGM) were applied on the etched surface, uniformly dispersed by compressed air blast for 2–5 s, and then cured for 20 s. Composite resin (Opus Bulk Fill Flow APS; FGM) was then injected into the root canal and cured to create a composite resin post. Finally, a matrix band was applied, and the tooth crown was reconstructed with the same composite resin used for the composite post such that the clinical crown height was 4 mm above the CEJ.

#### 2.3.2 Group 2: Prefabricated Glass Fiber Post (Whitepost)

**Figure 4:**
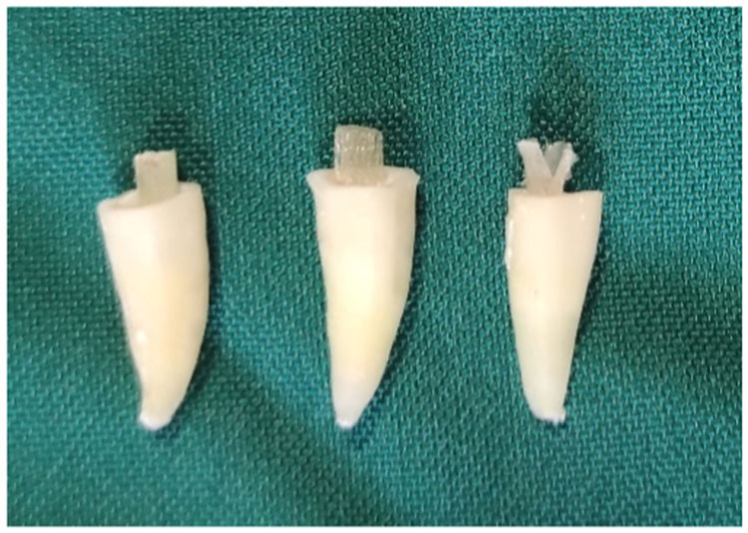
(Left) Whitepost, (middle) Ribbond, and (right) Interlig post.

A #3 fiber post (Whitepost; FGM) with a 2‐mm diameter was cut into a 5‐mm piece with a diamond bur and high‐speed handpiece under high water pressure and then cleaned with alcohol. Enamel, dentin, and root canal space were etched for 15 s as mentioned for Group 1. After washing and drying the root canal space, two layers of universal bonding (Ambar Universal; FGM) were applied to the root canal and cured. Next, flowable composite resin (Opus Bulk Fill Flow APS; FGM) and the post were introduced into the canal and cured. In the next step, the tooth was reconstructed as in Group 1 (Figure 4).

#### 2.3.3 Group 3: Customized Glass Fiber Post (Interlig)

Etching and bonding were performed as explained for Group 1. Next, the tip of the flowable composite resin applicator was inserted into the root canal by 2–3 mm, and composite resin was injected. Two separate tapes of 5‐mm Interlig fiber were cut with 2 mm width, placed in the root canal, and cured. Care was taken to maintain the fiber post 2 mm above the CEJ since 3 mm of the fiber post was required within the root canal, and 2 mm height was required out of the canal to serve as a core. Finally, the tooth crown was reconstructed as explained for Group 1 (Figure 4).

#### 2.3.4 Group 4: Customized Polyethylene Fiber Post (Ribbond)

Etching and bonding were performed as in Group 1. The tip of the Opus Bulk Fill Flow APS (FGM) composite resin applicator was inserted into the root canal by 2–3 mm, and composite resin was injected; 10 mm of the Ribbond fiber with 2 mm width was cut. Then, the fiber post was placed on a mixing pad and coated with resin (Margin Bond; Coltene, Germany). The excess resin was removed by squeezing the Ribbond post with tweezers. The fiber post was coated with two layers of the bonding agent, placed in the root canal, and cured. Care was taken to maintain the fiber 2 mm above the CEJ since 3 mm of the fiber post was required within the root canal, and 2 mm height was required out of the canal to serve as a core. Care was taken not to contaminate the fiber post. Finally, the tooth crown was reconstructed as explained in Group 1 (Figure 4).

### 2.4 Crown Rehabilitation

A groove with a standard length matching the dimensions of the indenter was created on the palatal surface of the restoration in all specimens. After restoration, all the specimens were polished with a composite resin polishing bur and high‐speed handpiece under water coolant and incubated at 37°C for 24 h (LTE IP60; Scientific Ltd., UK). The teeth were subsequently mounted in acrylic resin blocks such that 1 mm of the cervical part of the root remained exposed. To simulate the oral environment, the specimens underwent thermocycling (Neomo, Iran) for 5000 cycles in water baths between 5°C and 55°C with a dwell time of 30 s and a transfer time of 10 s.

### 2.5 Fracture Resistance and Fracture Strength

**Figure 5:**
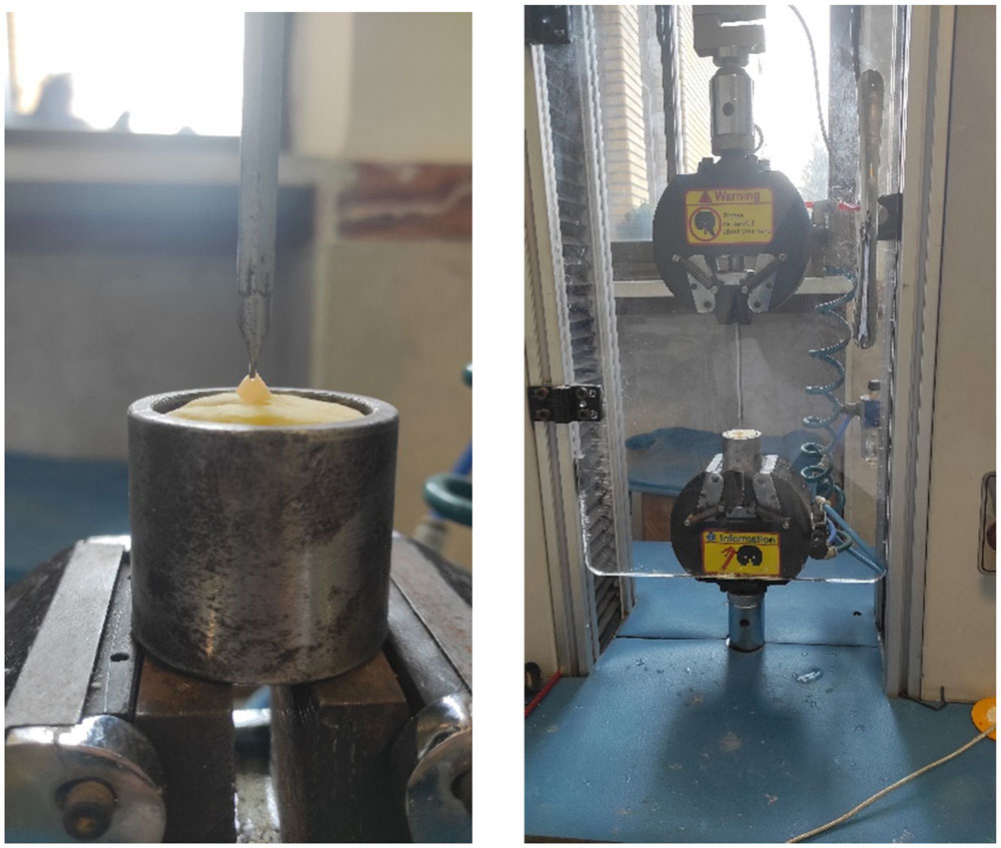
Specimens in a universal testing machine.

To measure the fracture resistance, the teeth were fixed to the jig of a universal testing machine (KOOPA, TB‐5T, Iran), and increasing force was applied at the midpalatal level with a crosshead speed of 0.5 mm/min at a 148° angle until fracture (Figure 5).

The force insertion angle is 135° in permanent teeth to simulate the occlusal forces applied to maxillary incisors in Class I occlusion. Since primary incisors are straight, Mangoush et al. (2017) suggested that this angle should be 148°. The fracture resistance was recorded in Newtons (N). By dividing the fracture resistance by the bonding surface area (mm^2^), the fracture strength of the samples was calculated in megapascals (MPa or N/mm^2^).

### 2.6 Fracture Mode

The fracture mode was also assessed. Fractures above the CEJ were restorable and were considered to be favorable. Nonfavorable fractures were defined as fractures below the CEJ that were not restorable (Mehra et al. 2016).

### 2.7 Statistical Analysis

Data were statistically analyzed using SPSS 20 (SPSS Inc., IL, USA) by one‐way ANOVA, *χ* ^2^ test, and the post hoc Tukey test. The level of significance was set at 0.05.

## 3 Results

Table 1 presents the fracture strength and fracture resistance of the four groups.

**Table 1:** Fracture strength and fracture resistance of the four groups.

Group	Fracture resistance (N)			Fracture strength (MPa)		
	Mean ± SD	Minimum–maximum	*p* value	Mean ± SD	Minimum–maximum	*p* value
Composite resin post	552.94 ± 149.76	370.95–884.91	0.096	22.45 ± 5.06	16.12–34.55	0.000
Whitepost	678.53 ± 168.42	372.06–916.75		33.10 ± 8.5	17.35–45.16	
Interlig post	659.85 ± 153.9	434.07–936.86		30.20 ± 7.33	22.12–46.73	
Ribbond post	669.03 ± 136.07	435.75–871.5		32.61 ± 5.73	20.10–42.18	

Abbreviation: SD, standard deviation.

**Chart 1:**
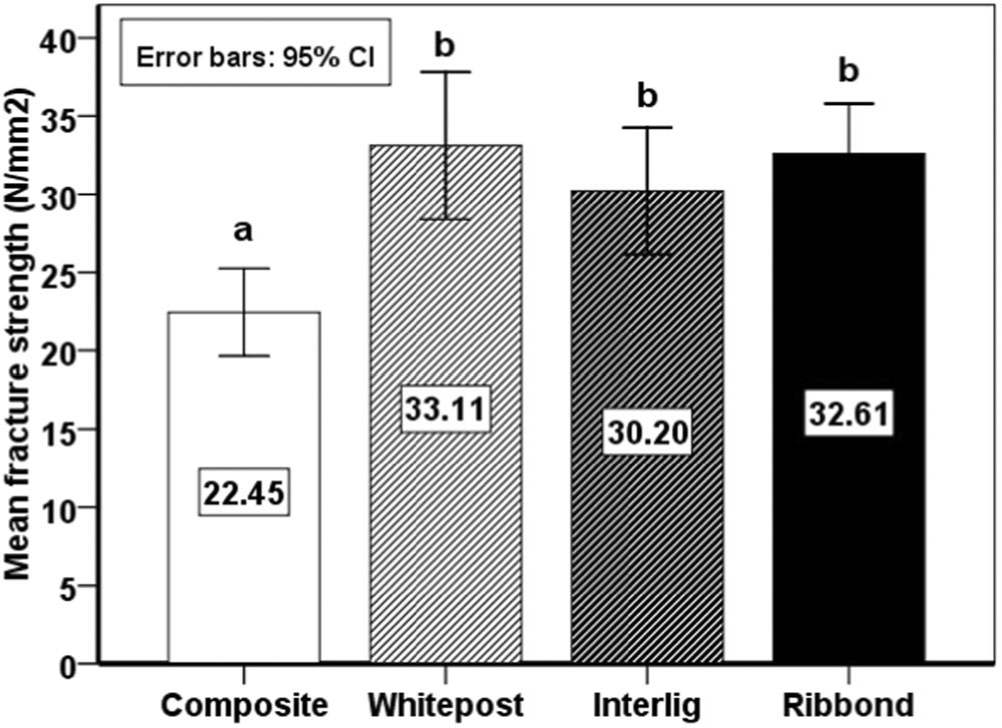
Pairwise comparisons of the mean fracture strength (N/mm^2^) of the study groups (different letters indicate the presence of statistically significant differences).

No significant difference was found in fracture resistance among the four groups (*p* = 0.096). One‐way ANOVA showed a statistically significant difference in fracture strength among the four groups (*p* = 0.000). Thus, pairwise comparisons were performed by the Tukey test, which showed that the fracture strength of the composite resin post group was significantly lower than that in the remaining three groups (*p* < 0.05). No other significant differences were found between the groups (Chart 1).

No significant difference was found among the four groups in the fracture mode (*p* > 0.05; Table 2). In all four groups, the frequency of favorable fractures was more than unfavorable fractures.

**Table 2:** Fracture modes in the study groups (*n* = 15).

Group	Above the CEJ (favorable)	Below the CEJ (unfavorable)	*p* value
Composite resin post	8	7	0.241
Whitepost	10	5	
Interlig post	8	7	
Ribbond post	11	4	

## 4 Discussion

In the past, extraction and replacement with fixed or removable partial dentures was the only treatment option for severely damaged teeth, which would cause problems for gingival health and was not feasible in children due to their poor cooperation. Pulpectomy and placement of intracanal posts are currently the preferred options for the reconstruction of severely damaged primary teeth (Arumugam and Sundaramurthy 2021). Intracanal posts are used to provide retention for the core to replace the lost coronal structure of the tooth and maintain the final restoration without compromising the apical seal of endodontic filling material. Therefore, it is important to select a post system that provides maximum retention by removing a minimum amount of tooth structure (Malakar, Tripathi, and Rahat 2019). Selecting the right intracanal post among the wide variety of materials available in the market is a challenging task for pediatric dentists (Arumugam and Sundaramurthy 2021).

The current study compared the fracture strength of four different intracanal posts, including a composite resin post, Whitepost, Interlig (customized glass fiber post), and Ribbond (customized polyethylene fiber post). The mode of fracture was also visually assessed in all specimens.

In the present study, the fracture strength of the composite resin post group was significantly lower than that of other groups (*p* = 0.00). The present results are consistent with the findings of Jurema et al. (2022), Srivastava and Thosar (2021), and Kadkhodaei et al. (2020), who showed the superiority of glass and polyethylene fiber posts to composite resin posts, due to better transfer of the applied stresses to a broader surface, and significantly increasing the fracture strength of teeth (Thakur and Ramarao 2019; Seraj et al. 2015).

The present results were not in line with the findings of Malakar, Tripathi, and Rahat (2019). They evaluated the fracture strength of different posts (composite resin, Ribbond, and Reforpost) in primary teeth. They found no statistically significant difference in fracture strength of the three materials. This difference between their results and the present findings may be due to using different materials, fewer thermal cycles, using Ribbond with the same length as the post space and not in two layers, and not using the same materials for cementation and reconstruction of the crown (absence of monoblock effect) in their study.

Thakur and Ramarao (2019) compared the fracture resistance of Ribbond (custom‐made polyethylene‐woven fiber post), Angelus (custom‐made glass fiber post), Reforpost (prefabricated glass fiber post), and prefabricated carbon post in extracted premolars. They also assessed the optimal post length in permanent teeth. For this reason, first, all the samples were divided into two groups with post lengths of 1/2 and 2/3 of the root canal length, and then they were assigned to four subgroups based on the type of post. They showed that in posts extending to 2/3 of the root length, prefabricated glass fiber posts yielded a significantly higher fracture resistance than other fiber posts. However, no significant difference was observed between other subgroups in posts extending to 1/2 of the root length. Differences between their results and the present findings may be attributed to differences in materials used, no conduction of thermocycling in their study, no evaluation of the bonded surface area of the samples, and simulation of periodontal ligament by using polytetrafluoroethylene strips. Also, the Angelus tape was used in one single layer. However, in the present study, Ribbond and Interlig tapes were used in two layers to simulate the clinical setting and eliminate the effect of this confounding factor. The application of two layers of Interlig fibers in the present study probably led to better stress distribution and increased the fracture strength in this group, and may explain why the fracture strength in this group was not significantly different from the fracture strength of the prefabricated group. In addition, the posts were extended by only 3 mm into the root canal system in the present study in order not to interfere with the eruption of permanent successors. Different results might have been obtained by increasing the length of the posts.

Mittal et al. (2018) studied the fracture strength of different posts (composite resin, customized quartz, and prefabricated glass) and found no significant difference in fracture strength of the samples. Difference between their results and the present findings can be attributed to using different materials and evaluation of all anterior teeth in their study (and not only canines). Canine teeth have a longer lifespan than primary incisor teeth, and they are among the last primary teeth to exfoliate in the maxilla. Additionally, primary canine teeth play a prominent role in maintaining the intercanine space, and in many cases, they may serve as a support for prosthetic appliances and space maintainers. Evaluation of only primary canine teeth in the present study eliminated the effect of many confounders, and can be considered as a strength of the present study.

In pediatric dentistry, provision of isolation for a long period of time is challenging due to young age and poor cooperation of children. Therefore, it is important to perform the procedures as fast as possible. The application of bulk‐fill flowable composite resin was another strength of the present study due to its injectability into the root canal and curing in 4‐mm‐thick increments in the root canal space. In addition, the bulk‐fill flowable composite resin was used for both cementation of the posts and forming the core in the present study to eliminate the confounding effect of using two different materials for this purpose and benefit from the monoblock effect, which could further reinforce the tooth structure (Thakur and Ramarao 2019).

It is worth mentioning that covering the tooth roots with silicone or wax before their mounting in acrylic resin may lead to root displacement during the loading process, which can cause inaccurate results. In addition, such periodontal ligament‐simulating materials have an elasticity different from that of the periodontium and cannot perfectly simulate the clinical setting (Salama et al. 2021). Thus, such materials were not used in the present study. In the current study, the teeth were cut 1 mm above the CEJ to simulate clinical conditions where the remaining tooth structure is reduced. Therefore, the compressive force is mainly borne by the post and core (Malakar, Tripathi, and Rahat 2019).

Thermocycling can affect the strength and durability of restorative materials in the oral cavity and decrease their fracture resistance (Mittal et al. 2018). Therefore, in the present study, all the specimens were subjected to 500 thermal cycles to increase the accuracy of the results and simulate the clinical setting.

The force was applied at a crosshead speed of 0.5 mm/min in a universal testing machine, and the fracture threshold was defined as the point at which the specimen could no longer withstand the applied force and fracture of the material, tooth, or root occurred (Malakar, Tripathi, and Rahat 2019).

Accurate measurement of the bonded surface area of the specimens was another strength of the present study, which was not considered in most previous studies, and despite using the term “fracture strength” in previous studies, they only measured the fracture resistance according to their description. Furthermore, studies that calculated the bonded surface area only examined the circular cross‐sectional area above the CEJ; none of them calculated the intracanal bonding area (Malakar, Tripathi, and Rahat 2019). This was made possible by taking standard photographs and using AutoCAD software in the present study.

It has been demonstrated that the root‐filling material does not interfere with the mechanical properties of the post system (Malakar, Tripathi, and Rahat 2019). Thus, Metapex was used in the present study for root canal filling. A review study also reported that many authors recommend using liners to better bond the post to the tooth structure (Nilavarasan et al. 2016). Various materials have been used as liners, including glass ionomer and polycarboxylate. In the present study, the post space was carefully separated from Metapex by applying 1 mm of polycarboxylate.

Some studies considered fractures in the cervical third of the tooth root as restorable. However, since crown lengthening surgery is not routinely performed in pediatric dentistry, fractures above the CEJ were classified as favorable fractures in the present study (Mittal et al. 2018). In the current study, the frequency of unfavorable fractures was 46% in the composite resin post group, 33% in the Whitepost, 46% in the Interlig, and 26% in the Ribbond group. The lowest rate of unfavorable fractures occurred in the Ribbond group, but no statistically significant difference was observed in this regard among the four groups. Although there was no group without posts in the present study, according to Sherfudhin et al. (2011), composite resin posts, glass, or polyethylene posts can decrease the frequency of unfavorable fractures.

In a study by Manns et al. (2022), the maximum chewing force in primary dentition was 301 N in the molar area and 150.42 N in the incisor area; these values were 421.34 N in the molar area and 208.17 N in the incisor area in mixed dentition period. In the oral environment, such forces are higher under physiological conditions, affecting the materials through continuous stress (Mittal et al. 2018). Obviously, the force that causes tooth fracture (post or core) is much higher than the force transmitted to the teeth during the mastication process. Force due to trauma can lead to tooth fracture (Malakar, Tripathi, and Rahat 2019). Since all specimens in the present study had a much higher fracture strength than the aforementioned values, it may be concluded that all the methods and materials used in this study are clinically acceptable under normal masticatory forces.

Mechanical retention may be required in some cases with composite resin posts, which are obtained by mushroom preparation of the coronal area of the root canal. It is worth mentioning that this preparation removes the tooth structure and is not favored. Also, due to the polymerization shrinkage of composite resin, the risk of retention loss of the post exists. The present results showed that the fracture strength of the Whitepost, Ribbond, and Interlig was significantly higher than that of composite resin post. As a result, customized and prefabricated polyethylene or glass fiber posts are preferred over composite resin posts. Among the remaining three groups, the highest fracture strength belonged to the Whitepost followed by the Ribbond and Interlig groups. However, the differences among these three groups were not statistically significant. Because of the lack of similar studies comparing different types of customized fiber posts, the present results could not be compared with any other study.

It is worth noting that the key factor in the success of customized ribbon fibers is placing the fibers at the closest distance to the cavity walls such that the fibers can absorb energy and increase resistance (Deliperi, Alleman, and Rudo 2017). When these fibers are used to strengthen the coronal structure of a tooth (e.g., in mesio‐occluso‐distal cavities), there is sufficient visibility and access to all the walls, and it is easy to place the fibers in close contact with the tooth walls. In the root canal of primary teeth, however, due to limited access and difficult handling, customized fibers cannot be perfectly adapted to the canal walls, and a larger gap may remain between the fibers and the tooth structure, which may be the reason for no superiority of customized posts to the prefabricated group. In addition, it may be stated that higher bond strength may be achieved by changing the orientation of fibers in the remaining coronal structure (e.g., on the floor or walls of the cavity in contact with the tooth structure). Additionally, it is hypothesized that if there is residual tooth structure, these fibers can better integrate with the tooth to distribute forces and create a wallpaper effect. Since the bond strength of the composite is potentially low due to the structure of primary teeth, using these fibers can strengthen the composite resin restorations, even in less damaged teeth.

Despite all the above, the clinical application of Interlig posts was highly challenging in the current study. Because of the preimpregnated nature of these fibers, it was difficult to place them in the root canal system. These fibers are light‐sensitive and thus their maintenance is challenging. Since the tape is long enough for use in several teeth, the fiber may harden and become brittle due to unwanted exposure to ambient light in each manipulation. Furthermore, placing the Interlig tape required great care because due to the different orientations of the strands (braided structure), the strands would separate and exceed the mesiodistal dimension of the crown in case of excessive manipulation. Interlig placement requires more time than other materials, which may be a drawback in pediatric dentistry. The Ribbond fiber also requires manual impregnation of the tape with the bonding agent. However, due to the interwoven nature of the strands, this tape is easier to work with and appears to be less technically sensitive. Whiteposts do not require special preparation steps, are easy to use, and yield favorable results.

This study had some limitations. It had an in vitro design; therefore, the measurements were static; also, the oral cavity has a dynamic environment. Finding sound teeth for the study was also challenging. Clinical studies are required to evaluate marginal discoloration, marginal adaptation, and post dislodgement. Also, fatigue tests should be performed in future studies.

## 5 Conclusion

Based on the present results, prefabricated glass fiber post (Whitepost), customized glass fiber post (Interlig), and customized polyethylene fiber post (Ribbond) showed a significantly higher fracture strength than the composite resin post. There was no significant difference among the groups regarding the fracture mode.

## Author Contributions

**Nilgoon Pasdar and Ghazaleh Ahmadizenouz:** conceptualization. **Faeze Behzadpour, Nilgoon Pasdar, and Ghazaleh Ahmadizenouz:** methodology. **Ali Bijani:** formal analysis. **Faeze Behzadpour:** writing–original draft preparation. **Nilgoon Pasdar and Ghazaleh Ahmadizenouz:** writing–review and editing. All authors have read and agreed to the published version of the manuscript.

## Conflicts of Interest

The authors declare no conflicts of interest.

## Data Availability

The data used to support the findings of the current study are available upon request from the corresponding author.
